# Healthcare-Associated COVID-19 across Five Pandemic Waves: Prediction Models and Genomic Analyses

**DOI:** 10.3390/v14102292

**Published:** 2022-10-18

**Authors:** Thomas Demuyser, Lucie Seyler, Rhea Buttiens, Oriane Soetens, Els Van Nedervelde, Ben Caljon, Jessy Praet, Thomas Seyler, Joost Boeckmans, Jessy Meert, Robin Vanstokstraeten, Helena Martini, Florence Crombé, Denis Piérard, Sabine D. Allard, Ingrid Wybo

**Affiliations:** 1 Department of Microbiology and Infection Control, Vrije Universiteit Brussel (VUB), Universitair Ziekenhuis Brussel (UZ Brussel), Laarbeeklaan 101, 1090 Brussels, Belgium; 2 Center for Neurosciences, Faculty of Medicine and Pharmacy, Vrije Universiteit Brussel (VUB), Laarbeeklaan 103, 1090 Brussels, Belgium; 3 Department of Internal Medicine and Infectious Diseases, Universitair Ziekenhuis Brussel (UZ Brussel), Vrije Universiteit Brussel (VUB), Laarbeeklaan 101, 1090 Brussels, Belgium; 4 Brussels Interuniversity Genomics High Throughput Core (BRIGHTcore), Universitair Ziekenhuis Brussel (UZ Brussel), Vrije Universiteit Brussel (VUB), Laarbeeklaan 101, 1090 Brussels, Belgium; 5 bioMérieux, Data Analytics, Keistraat 120, 9830 St-Martens-Latem, Belgium; 6 Field Epidemiologist; 7 Department of In Vitro Toxicology and Dermato-Cosmetology, Faculty of Medicine and Pharmacy, Vrije Universiteit Brussel (VUB), Laarbeeklaan 103, 1090 Brussels, Belgium; 8 Clinical Laboratory, Jessa Hospital, Stadsomvaart 11, 3500 Hasselt, Belgium

**Keywords:** healthcare-associated COVID-19, prediction modelling, genomic analysis

## Abstract

Background: Healthcare-associated SARS-CoV-2 infections need to be explored further. Our study is an analysis of hospital-acquired infections (HAIs) and ambulatory healthcare workers (aHCWs) with SARS-CoV-2 across the pandemic in a Belgian university hospital. Methods: We compared HAIs with community-associated infections (CAIs) to identify the factors associated with having an HAI. We then performed a genomic cluster analysis of HAIs and aHCWs. We used this alongside the European Centre for Disease Control (ECDC) case source classifications of an HAI. Results: Between March 2020 and March 2022, 269 patients had an HAI. A lower BMI, a worse frailty index, lower C-reactive protein (CRP), and a higher thrombocyte count as well as death and length of stay were significantly associated with having an HAI. Using those variables to predict HAIs versus CAIs, we obtained a positive predictive value (PPV) of 83.6% and a negative predictive value (NPV) of 82.2%; the area under the ROC was 0.89. Genomic cluster analyses and representations on epicurves and minimal spanning trees delivered further insights into HAI dynamics across different pandemic waves. The genomic data were also compared with the clinical ECDC definitions for HAIs; we found that 90.0% of the ‘definite’, 87.8% of the ‘probable’, and 70.3% of the ‘indeterminate’ HAIs belonged to one of the twenty-two COVID-19 genomic clusters we identified. Conclusions: We propose a novel prediction model for HAIs. In addition, we show that the management of nosocomial outbreaks will benefit from genome sequencing analyses.

## 1. Introduction

During the first wave of the pandemic, approximately 10 to 15% of hospitalized COVID-19 cases were hospital-acquired infections (HAIs) [1,2]; front-line healthcare workers (HCWs) also had an increased risk of acquiring COVID-19 by a factor of 3.4 compared with the general population [3,4]. However, HAIs in the first wave might not be representative of later phases of the pandemic. Indeed, initial HAIs may have been partly attributable to incorrect isolation procedures, the indistinct use of shared healthcare equipment, movements of infected personnel, and insufficient knowledge and awareness of viral transmission properties [5]. Knowledge of the characteristics of the virus increased dramatically thereafter. In addition to better infection control interventions, vaccines and non-pharmaceutical interventions were rolled out, with dramatic effects on viral transmission [6]. 

However, SARS-CoV-2 kept evolving. A few variants of concern (VOCs) have been related to more severe infections, high viral loads [7], vaccine escape, or increased transmissibility [8]. The rapid spread of the Delta and Omicron variants, and maybe other variants in the future, only stresses the importance of continuous epidemiological surveillance [9] and preparedness within hospitals is crucial. 

Many articles have described community-associated COVID-19 (CAI) patients from the beginning of the pandemic onwards [1,10]. Fewer have focused on HAIs, despite the burden and risks associated with HAIs [11]. We decided, therefore, to study our healthcare-associated cases (HAIs and aHCWs) using different approaches: clinical, epidemiological, and genomic [12,13,14].

## 2. Materials and Methods

### 2.1. Setting and Timeline

Our study was conducted in Belgium from 6 March 2020 until 2 March 2022, spanning 2 years of the SARS-CoV-2 pandemic at ‘Universitair Ziekenhuis Brussel’, a 721-bed Belgian tertiary care center. 

Infection control measures against SARS-CoV-2 in our hospital followed the national guidelines and changed over time. So did the testing strategy in our center: PCR tests became available in early March 2020; from 16 May 2020, all patients were screened with a PCR upon hospitalization; from 29 April 2021, an extra PCR screening of all patients was performed at day 4 and day 7 after admission and every week thereafter (until the end of the study period). 

Five different epidemic waves were identified by our national public health institute. The vaccine rollout started in our hospital in January 2021; the uptake was extremely high, reaching >95% of the hospital staff.

### 2.2. Definitions and Inclusions

A case of a CAI was an adult with a community-acquired infection admitted for >24 h. A healthcare-associated COVID-19 infection is a general term, and included hospitalized patients with a nosocomial COVID-19 infection and healthcare workers diagnosed with COVID-19 whilst in service (this does not indicate whether the infection was acquired in the hospital setting). A case of an aHCW was an ambulatory healthcare worker from our hospital with a diagnosis of COVID-19. A case of an HAI was an adult with a hospital-acquired COVID-19 infection during a hospitalization for another indication admitted >24 h following the COVID-19 diagnosis. To identify an HAI in our study, we used the European Centre for Disease Control (ECDC) case source definitions of an HAI, as described in Table 1 [12]. A ‘symptomatic’ HAI was a patient in whom one or more of the World Health Organization-defined criteria for a severe acute respiratory infection (SARI) were present at a COVID-19 diagnosis (fever ≥ 37.8 °C, cough, and shortness of breath).

All the subjects included in the study (Table 2) had a COVID-19 infection confirmed by a PCR. Data from all HAI patients and from a random selection of all hospitalized CAIs were de-identified and anonymized. We used the CAI patients as a comparison group for the first part of our study. aHCWs were identified from the infection control database.

### 2.3. Objectives and Study Design

The objective of this study was to better characterize HAIs in our university hospital. For this purpose, we combined a multiple logistic regression on retrospective data with genomic analyses. As such, we could identify the predictive factors associated with having an HAI versus a CAI. To give a better idea of the distribution of the HAI and CAI cases over time, we computed all of our cases on epidemiological curves alongside the genomic trees. We also showed the added value of the genomic cluster analysis to the ECDC source definitions.

### 2.4. Laboratory: Inclusion of Samples for Genetic Sequencing and Genomic Cluster Analyses

Nasopharyngeal samples from HAIs and aHCWs were systematically stored at −80 °C. All samples with sufficiently high (cycle threshold value ≤ 25) viral loads and enough remaining volume were included in the whole genome sequencing (WGS) analysis of the current study. 

We adapted a SARS-CoV-2 WGS protocol [15]. Amplicon libraries were sequenced using MinION flow cells (Oxford Nanopore Technologies, Oxford, UK). Genomes were assembled with a reference-based assembly and an in-house bioinformatic pipeline with a 300 × minimum coverage cut-off for any region of the genome. Consensus FASTA sequences were generated [16]. The gene sequences were uploaded onto the Global Initiative on Sharing All Influenza Data (GISAID) platform (see web-only Supplementary Table S1) [17].

The WGS data were processed with the SARS-CoV-2 plug-in of BioNumerics v.8.1 (Applied Maths, Biomérieux, Sint-Martens-Latem, Belgium). The sub-sequences of the Wuhan-Hu-1 (NC 04551219) reference genome were used as the reference sequences for a BLAST search [18]. After the extraction, these sub-sequences were screened for single nucleotide polymorphisms (SNPs). Fifteen entries with an incomplete SNP character set were excluded from the further analysis (all included genomes are depicted in Supplementary Table S1). 

A similarity matrix was then calculated based on the remaining SNP experiments and minimal spanning trees (MSTs) were constructed. Genomic clusters were defined as genomes with a difference of ≤2 SNPs and were marked with a grey contour. Therefore, every visually distinct gray contour represented a separate genomic cluster. Twenty-two clusters involving at least one HAI patient were identified. We looked at the HAI within each ECDC category and checked if they were part of a cluster or not.

### 2.5. Statistical and Epidemiological Analyses

We used descriptive statistics to report on the demographics as well as the clinical and laboratory data of the patients and then compared the continuous and categorical variables between the HAI and CAI patients. To obtain numerically comparable groups for the comparative analyses, a random selection of the CAI patients was made. For the continuous data, the median values and interquartile ranges were shown and a univariate statistical analysis was performed by an unpaired *t*-test or a Mann–Whitney test (depending on the normal distribution of the data). The categorical data were expressed in absolute numbers and percentages; the univariate statistical analysis was performed by a Fisher’s exact test. A *p*-value < 0.05 was considered to be statistically significant. 

We used the parameters shown to be statistically significant in the univariate analysis to perform the multiple logistic regression analyses by means of GraphPad Prism 8.4.3 software (San Diego, CA, USA). Positive and negative predictive values were calculated as well as the area under the ROC curves.

We showed all of the cases over time on epidemiological curves, hereafter called epicurves, using STATA scripts and Excel software and reported them by the week of diagnosis (Supplementary Table S4). All of the patients mentioned in Table 2 were included in the epicurves. We showed them alongside the minimal spanning trees.

The hospital wards were anonymized; ward names were replaced by numbers, themselves corresponding with the floor on which the ward could be found.

## 3. Results

**Figure 1:**
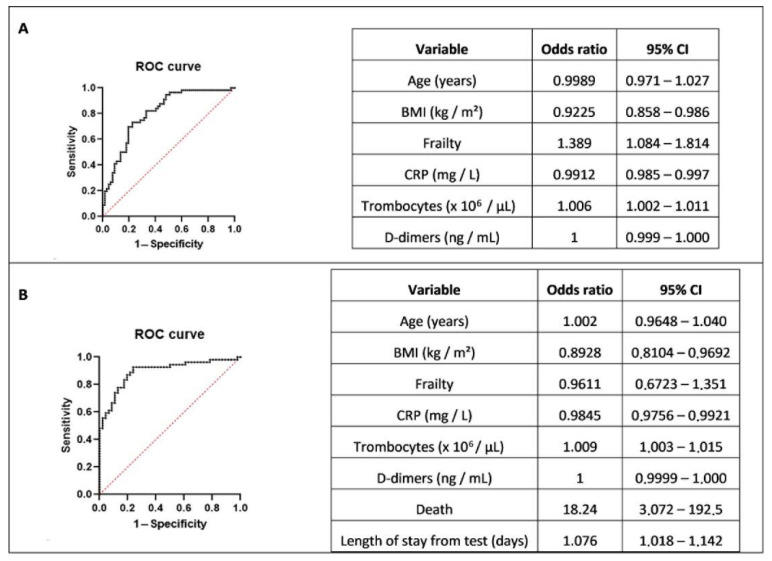
Multiple logistic regression modelling of HAI versus CAI patients. (**A**) Multiple logistic regression without death and length of stay variables. (**B**) Multiple logistic regression with death and length of stay variables.

The results of the univariate analyses of HAIs versus CAIs are described in Supplementary Table S2. Leaving comorbidities and symptoms aside for the multiple logistic regression, we reported the results of two models; one without and one with the inclusion of death and length of stay as variables, as shown in Figure 1A,B, respectively. The odds ratios reflected the effect of that parameter on the probability that a patient had an HAI; the BMI, frailty index, C-reactive protein (CRP), and thrombocyte count at the COVID-19 diagnosis of the patient remained significant at predicting an HAI. Similarly, the outcome measures ‘death’ and ‘length of stay from diagnosis’ were significantly correlated with having an HAI. With the results presented in Figure 1A, we obtained a positive predictive value of 74.0% and a negative predictive value of 74.0%; the area under the receiver operator curve (ROC) was 0.81. With the results presented in Figure 1B, we obtained a positive predictive value of 83.6% and a negative predictive value of 82.2%; the area under the ROC was 0.89.

**Figure 2:**
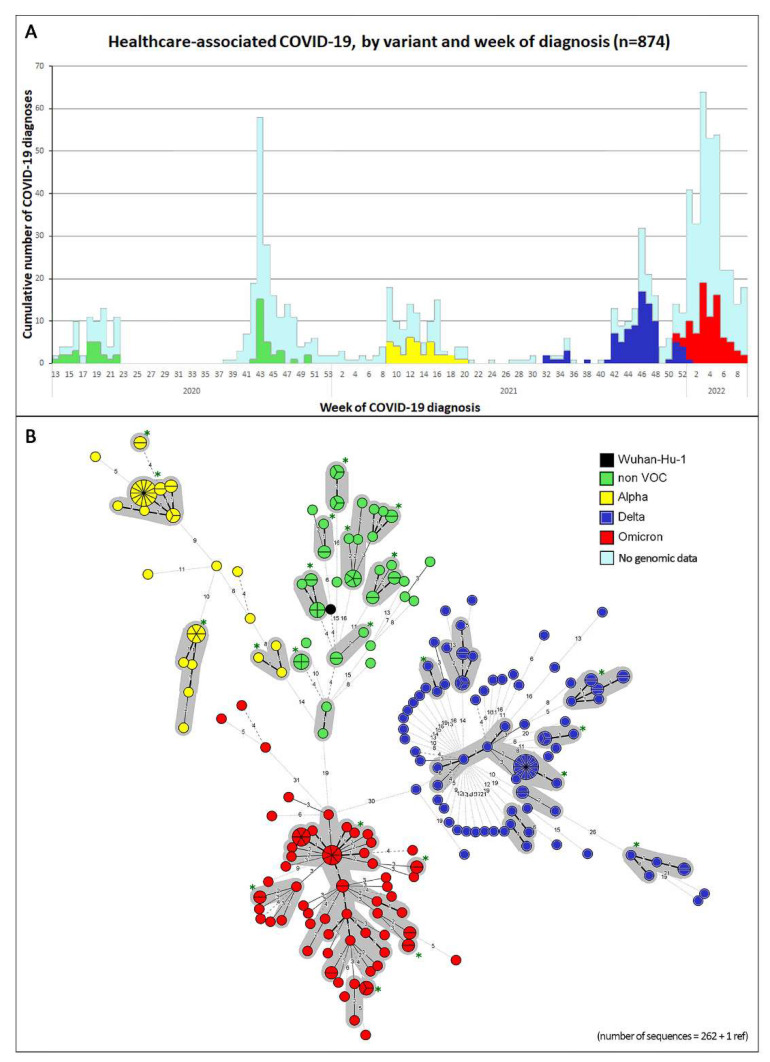
Epicurve and MST of healthcare-associated COVID-19 infections by WHO SARS-CoV-2 class and week of diagnosis. (**A**) Epicurve of our cases showing all healthcare-associated COVID-19 infections (HAI and aHCW) with SARS-CoV-2 WHO classes by color. Patients from whom no genomic data were available are marked in light blue. (**B**) MST (minimal spanning tree) of SARS-CoV-2 genomes of the patients whose samples were available for sequencing. The black genome represents the reference genome Wuhan-Hu-1 [18]. The size of the circles is proportional to the number of cases. Genomes that differed by ≤2 SNPs were clustered using the grey contours (HAI and aHCW together). Green asterisks are used to show the 22 clusters that contained at least 1 HAI case.

Figure 2A shows the 269 HAI cases and the 605 aHCWs taken together (*n* = 874) over time on the epicurve, color-coded according to the viral strain. Figure 2B shows the MST of the sequenced samples (*n* = 262), color-coded by the type of SARS-CoV-2 strain. The genomic clusters are highlighted by the contoured circles in grey. 

**Figure 3:**
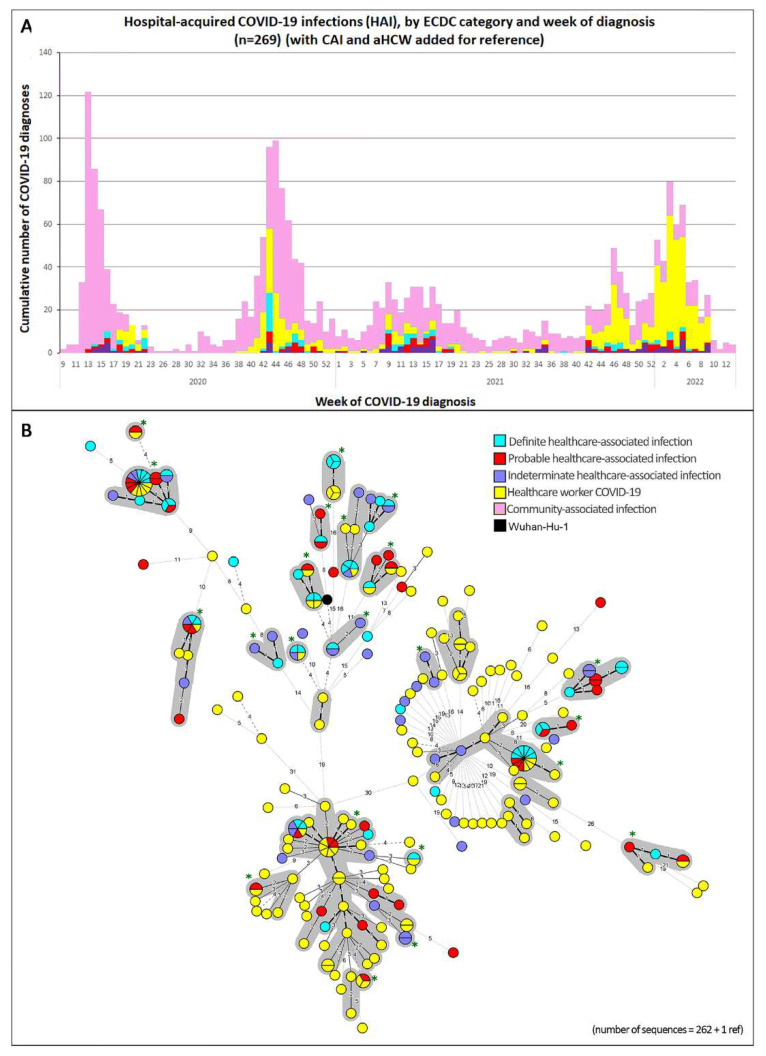
Epicurve and minimum spanning tree (MST) of hospital-acquired infections (HAIs) (*n* = 269) by ECDC case source definition and week of diagnosis (aHCWs and CAIs were included for reference). (**A**) Epicurve of our cases showing all HAIs by ECDC categories and by week. CAIs (pink) and aHCWs (yellow) are shown for reference. (**B**) MST (minimal spanning tree) of SARS-CoV-2 genomes of the patients whose samples were available for sequencing. The black genome represents the reference genome Wuhan-Hu-1 [18]. The size of the circles is proportional to the number of cases. Genomes that differed by ≤ 2 SNPs were clustered using the grey contours (HAI and aHCW together). Green asterisks are used to show the 22 clusters that contained at least 1 HAI case.

We classified our hospitalized HAI patients into subgroups according to the case source definition used by the ECDC (Supplementary Table S3). Figure 3 shows our 269 HAI cases on an epicurve (Figure 3A) and in an MST (Figure 3B), color-coded by the ECDC classification; the grey contours highlight the clusters. The proportions of samples in each ECDC category, part of any cluster, were 70.3, 87.8, and 90% for each category, in increasing order of certainty.

## 4. Discussion

Our study is one of few addressing the complex issue of healthcare-associated COVID-19 infections comprehensively and across the whole pandemic (up to March 2022). We describe a novel approach in the differentiation between HAIs and CAIs. We obtained a positive predictive value (PPV) of 83.6% and a negative predictive value (NPV) of 82.2%, when computing death and LOS into the model. From the genomic analyses we learned that infections amongst HAIs and aHCWs were successively due to the pre-VOC virus, and then the Alpha, Delta, and Omicron VOCs; this was consistent with the SARS-CoV-2 variant waves observed in the CAIs in Belgium. This finding may seem logical, but it is still noteworthy: having sicker patients (HAIs) or well-vaccinated aHCWs did not change the type of virus with which they were infected. We found that larger proportions of patients who had been classified as having an HAI in the higher levels of certainty (using the ECDC rules) were part of one of the twenty-two COVID-19 genomic clusters (90.0%, 87.8%, and 70.3%). Whether a patient belonged to a cluster or not added an extra dimension to the ECDC definitions and also confirmed the validity of the ECDC criteria. 

Our proportion of HAIs seemed comparable with that of other centers [1,2]. It was stable across the first two waves (10.5–12.6%) and increased toward the third wave (22.5%) despite more control measures being in place. At a time when the vaccines were not available, this probably reflected the dominance of the more infectious Alpha VOC. Thereafter, the proportion of HAIs decreased with the vaccine rollout (14.1% in wave four). The occurrence of the even more contagious Omicron variant explained the next rise in (mainly asymptomatic) HAIs (24.9 % in wave five). Differences between HAI and CAI patients might be due to the timing of COVID-19 diagnoses [10] and general patient characteristics [11]. However, even if taking those arguments into account, our second regression model (Figure 1B) may be useful for describing the dataset of a hospital retrospectively (‘death’ and ‘length of stay’ information can only be collected retrospectively). Of course, a detection tool is most useful if it can predict if a patient has an HAI; in that case, our first regression model (Figure 1A) may be best suited because a lower BMI, higher frailty score, lower CRP, and higher thrombocyte count were linked with having an HAI in our dataset. This is a first step toward building a tool for the detection and diagnosis of HAIs. Our findings will have to be replicated first, completed with other factors, and then validated to become applicable. 

Until wave 3, many clusters were identified on the epicurve (Figure 2 and Figure 3) as well as by the grey contours on the corresponding MSTs. A few of the clusters were large, stressing once again the extreme infectiousness of this agent at a time without vaccine coverage. During wave 5, however, the clusters were much more difficult to distinguish; many more infections (amongst aHCWs or HAIs) were distinct from each other. There are many possible explanations for this. The later VOCs were much more contagious. The higher contagiousness of the Omicron virion may also be associated with fewer mutations in the virus [19] as it has less time to mutate between hosts. The lower virulence of the Omicron variant may be another factor; patients were allowed to leave hospital much faster and thus interrupt the transmission chains earlier. Furthermore, if patients (HAIs or aHCWs) achieved a lower viral load quicker than in earlier waves, their samples could not be sequenced; this may have led to a selection bias away from the clusters. In wave 5, we also saw many more cases of aHCWs compared with the earlier waves. Many of them probably acquired their infection outside the hospital. Public health measures were less strict in Belgium at that time. 

Generalizing the results of a single-center study is never easy, but the advantage of a monocentric study in this context is the more uniform management of infection prevention strategies (vaccination, PPE, isolation, and screening strategies, for example), thus limiting the introduction of other biases. Another important limitation was the proportion of samples that we were able to sequence. Only samples with a high enough viral load could be sequenced. This reflected the technical reality worldwide. We fully acknowledge that this may have introduced an element of selection bias, possibly toward other (and maybe more) pathogenic viruses, but we still think that our results gave a very good idea of the genomic dynamics amongst HAIs and aHCWs in a large academic hospital and in a real-life setting. 

The issue of HAIs will sadly remain topical until we manage to improve their management by combining several approaches. Our study contributes to the body of knowledge toward that goal.

## Figures and Tables

**Table 1:** ECDC case source definitions of COVID-19 [12].

Case Source COVID-19	Definition
CAI	Symptoms or sample present on admission or with onset on day 1 or 2 after admission (or on days 3–7 with a strong suspicion of community transmission).
Indeterminate HAI	Symptom onset or sample present on days 3–7 after admission with insufficient information on the source of infection to assign to another category.
Probable HAI	Symptom onset or sample present on days 8–14 after admission (or on days 3–7 and a strong suspicion of healthcare transmission).
Definite HAI	Symptom onset or sample present on day > 14 after admission.

Different categories of community (CAI)- or healthcare (HAI)-associated COVID-19 infections with an increasing likelihood of a healthcare-associated infection from top to bottom. A ‘sample’ is defined as a positive laboratory test result confirming an acute COVID-19 infection.

**Table 2:** An overview of the patients and healthcare workers included in this study.

COVID-19 Wave in Belgium	Date	Hospitalized Patients			aHCW
		CAI	HAI		
		Symptomatic (Random Selection for Comparison)	Total (% of CA + HA)	Symptomatic (% of Total HA)	Number
Wave 1	2 March 2020–7 June 2020	365 (37)	43 (10.5)	36 (83.7)	29
Between waves 1 and 2	8 June 2020–6 September 2020	39 (3)	0 (0.0)	NA	0
Wave 2	7 September 2020–3 January 2021	409 (41)	59 (12.6)	34 (57.6)	132
Wave 3	4 January 2021–4 July 2021	320 (32)	72 (22.5)	48 (66.7)	51
Between waves 3 and 4	5 July 2021–31 October 2021	151 (15)	18 (10.7)	8 (44.4)	25
Wave 4	1 November 2021–26 December 2021	158 (16)	26 (14.1)	16 (61.5)	90
Wave 5	27 December 2021–6 March 2022	154 (15)	51 (24.9)	23 (45.1)	278
Total		1596 (159)	269 (14.4)	165 (61.3)	605

HAI—healthcare-associated COVID-19 infection; CAI—community-associated COVID-19 infection; aHCW—ambulatory healthcare workers with a COVID-19 infection.

## Data Availability

The data of patients included in this manuscript are considered sensitive and will not be shared. The study methods and statistical analyses are described in detail in the Methods section. The virus genomic data are shared on GISAID (see Supplementary Table S1).

## Supplementary Materials

The following supporting information can be downloaded at: https://www.mdpi.com/article/10.3390/v14102292/s1, Table S1: Detailed information on included genomes; Table S2: Demographic, clinical, and laboratory data of symptomatic healthcare-associated versus community-associated hospitalized COVID-19 patients (UZ Brussel, 2020–2022): univariate analysis; Table S3: Hospital-acquired infections according to the ECDC case source definition, Table S4: STATA scripts.
