# Supplementary material for: Healthcare-Associated COVID-19 across Five Pandemic Waves: Prediction Models and Genomic Analyses
Source: Viruses. 2022 Oct 18;14(10):2292. doi: 10.3390/v14102292 (PMC9607632; doi:10.3390/v14102292)
Supplement: Supplementary file 1 [file viruses-14-02292-s001.zip › Supplementary Table S1.pdf]

Supplementary Table S1

Detailed information on included genomes

Patient / HCW	GISAID Accession ID	Sample date	Pangolineage	WHO class	Ward	HAI or CAI
1	EPI_ISL_3138679	2020-03-27	B.38	non-VOC	1A	Probable HAI
2	EPI_ISL_3138680	2020-04-01	B.1	non-VOC	1B	Definite HAI
3	EPI_ISL_3138681	2020-04-02	B.1	non-VOC	1B	Indeterminate HAI
4	EPI_ISL_3138682	2020-04-07	B.38	non-VOC	1A	Indeterminate HAI
5	EPI_ISL_3138683	2020-04-10	B.38	non-VOC	1A	Probable HAI
6	EPI_ISL_3138684	2020-04-14	B.38	non-VOC	1B	Definite HAI
7	EPI_ISL_3138685	2020-04-14	B.1	non-VOC	1A	Indeterminate HAI
8	EPI_ISL_3138656	2020-04-14	B.1	non-VOC	2A	Indeterminate HAI
9	EPI_ISL_3138739	2020-04-27	B.1	non-VOC	2A	Definite HAI
10	EPI_ISL_3138686	2020-04-27	B.1	non-VOC	2A	Probable HAI
11	EPI_ISL_3138734	2020-05-01	B.1.221	non-VOC	Other	Healthcare worker
12	EPI_ISL_3138687	2020-05-02	B.1	non-VOC	2A	Probable HAI
13	EPI_ISL_3138688	2020-05-03	B.1	non-VOC	2A	Probable HAI
14	EPI_ISL_3138689	2020-05-04	B.1	non-VOC	3AB	Healthcare worker
15	EPI_ISL_3138690	2020-05-05	B.1	non-VOC	2A	Healthcare worker
16	EPI_ISL_3138692	2020-05-06	B.1	non-VOC	3AB	Definite HAI
17	EPI_ISL_3138691	2020-05-07	B.1	non-VOC	3AB	Healthcare worker
18	EPI_ISL_3138693	2020-05-10	B.1	non-VOC	3AB	Definite HAI
19	EPI_ISL_3138694	2020-05-12	B.1	non-VOC	3AB	Probable HAI
20	EPI_ISL_3138695	2020-05-16	B.1	non-VOC	3AB	Healthcare worker
21	EPI_ISL_3138696	2020-05-20	B.1	non-VOC	3AB	Healthcare worker
22	EPI_ISL_3138697	2020-05-25	B.1	non-VOC	2B	Definite HAI
23	EPI_ISL_3138659	2020-10-15	B.1.1.269	non-VOC	2B	Indeterminate HAI
24	EPI_ISL_3138733	2020-10-20	B.1.1.44	non-VOC	2B	Definite HAI
25	EPI_ISL_3138660	2020-10-20	B.1.1.44	non-VOC	2C	Definite HAI
26	EPI_ISL_3138737	2020-10-21	B.1.221	non-VOC	2C	Definite HAI
27	EPI_ISL_3138736	2020-10-21	B.1.221	non-VOC	2C	Definite HAI
28	EPI_ISL_3138738	2020-10-21	B.1.1	non-VOC	2B	Healthcare worker
29	EPI_ISL_3138661	2020-10-21	B.1.160	non-VOC	2A	Indeterminate HAI
30	EPI_ISL_3138735	2020-10-21	B.1.221	non-VOC	2C	Definite HAI
31	EPI_ISL_3138663	2020-10-22	B.1.160	non-VOC	2A	Healthcare worker
32	EPI_ISL_3138662	2020-10-22	B.1.160	non-VOC	2A	Indeterminate HAI
33	EPI_ISL_3138664	2020-10-23	B.1.221	non-VOC	2D	Healthcare worker
34	EPI_ISL_3138665	2020-10-25	B.1.1	non-VOC	2B	Indeterminate HAI
35	EPI_ISL_3138667	2020-10-25	B.1.221	non-VOC	2C	Healthcare worker
36	EPI_ISL_3138666	2020-10-25	B.1.160	non-VOC	2B	Definite HAI
37	EPI_ISL_3138670	2020-10-26	B.1.177.77	non-VOC	2B	Healthcare worker
38	EPI_ISL_3138669	2020-10-26	B.1.1	non-VOC	2B	Healthcare worker
39	EPI_ISL_3138671	2020-10-27	B.1.177	non-VOC	2A	Healthcare worker
40	EPI_ISL_3138672	2020-11-07	B.1.160	non-VOC	3AB	Definite HAI
41	EPI_ISL_3138673	2020-11-08	B.1.160	non-VOC	3AB	Definite HAI
42	EPI_ISL_3138675	2020-11-23	B.1.177.77	non-VOC	2B	Definite HAI
43	EPI_ISL_3138676	2020-12-07	B.1.177.77	non-VOC	3AB	Probable HAI
44	EPI_ISL_3138677	2020-12-08	B.1.221	non-VOC	5A	Probable HAI

45	EPI_ISL_3138678	2020-12-13	B.1.177.77	non-VOC	2C	Healthcare worker
46	EPI_ISL_3138699	2021-03-01	B.1.1.7	Alpha	4A	Definite HAI
47	EPI_ISL_3138698	2021-03-01	B.1.1.7	Alpha	4A	Probable HAI
48	EPI_ISL_3138701	2021-03-02	B.1.1.7	Alpha	4A	Indeterminate HAI
49	EPI_ISL_3138702	2021-03-03	B.1.1.7	Alpha	4A	Probable HAI
50	EPI_ISL_3138700	2021-03-06	B.1.1.7	Alpha	4A	Probable HAI
51	EPI_ISL_3138703	2021-03-08	B.1.1.7	Alpha	4A	Healthcare worker
52	EPI_ISL_3138706	2021-03-09	B.1.1.7	Alpha	4A	Definite HAI
53	EPI_ISL_3138704	2021-03-09	B.1.1.7	Alpha	4A	Definite HAI
54	EPI_ISL_3138705	2021-03-09	B.1.1.7	Alpha	4A	Definite HAI
55	EPI_ISL_3138707	2021-03-14	B.1.1.7	Alpha	4A	Indeterminate HAI
56	EPI_ISL_3138708	2021-03-15	B.1.1.7	Alpha	2A	Definite HAI
57	EPI_ISL_3138709	2021-03-16	B.1.1.7	Alpha	2A	Indeterminate HAI
58	EPI_ISL_3138710	2021-03-18	B.1.1.7	Alpha	Other	Healthcare worker
59	EPI_ISL_3138711	2021-03-21	B.1.1.7	Alpha	2A	Healthcare worker
60	EPI_ISL_3138712	2021-03-22	B.1.1.7	Alpha	2A	Healthcare worker
61	EPI_ISL_3151138	2021-03-23	B.1.1.7	Alpha	1C	Definite HAI
62	EPI_ISL_3138714	2021-03-23	B.1.1.7	Alpha	1C	Probable HAI
63	EPI_ISL_3138713	2021-03-23	B.1.1.7	Alpha	1C	Probable HAI
64	EPI_ISL_3138715	2021-03-24	B.1.1.7	Alpha	1C	Probable HAI
65	EPI_ISL_3138716	2021-03-24	B.1.1.7	Alpha	3AB	Healthcare worker
66	EPI_ISL_3138717	2021-03-26	B.1.1.7	Alpha	1C	Healthcare worker
67	EPI_ISL_3138718	2021-03-26	B.1.1.7	Alpha	2B	Definite HAI
68	EPI_ISL_3138720	2021-03-28	B.1.1.7	Alpha	2B	Definite HAI
69	EPI_ISL_3138719	2021-03-28	B.1.1.7	Alpha	2B	Definite HAI
70	EPI_ISL_3138721	2021-03-29	B.1.1.7	Alpha	2B	Healthcare worker
71	EPI_ISL_3138740	2021-03-29	B.1.1.7	Alpha	2B	Healthcare worker
72	EPI_ISL_3138723	2021-03-30	B.1.1.7	Alpha	1C	Indeterminate HAI
73	EPI_ISL_3138722	2021-03-30	B.1.1.7	Alpha	3C	Definite HAI
74	EPI_ISL_3138724	2021-04-06	B.1.1.7	Alpha	3C	Indeterminate HAI
75	EPI_ISL_3138725	2021-04-06	B.1.1.7	Alpha	3C	Indeterminate HAI
76	EPI_ISL_3138726	2021-04-17	B.1.1.7	Alpha	3AB	Definite HAI
77	EPI_ISL_3138729	2021-04-18	B.1.1.7	Alpha	3AB	Indeterminate HAI
78	EPI_ISL_3138730	2021-04-18	B.1.1.7	Alpha	3AB	Probable HAI
79	EPI_ISL_3138728	2021-04-18	B.1.1.7	Alpha	3AB	Indeterminate HAI
80	EPI_ISL_3138727	2021-04-18	B.1.1.7	Alpha	3AB	Probable HAI
81	EPI_ISL_3138731	2021-04-19	B.1.1.7	Alpha	3AB	Healthcare worker
82	EPI_ISL_3138657	2021-04-23	B.1.1.7	Alpha	3AB	Definite HAI
83	EPI_ISL_3138658	2021-04-26	B.1.1.7	Alpha	3AB	Healthcare worker
84	EPI_ISL_3138732	2021-04-26	B.1.1.7	Alpha	3AB	Healthcare worker
85	EPI_ISL_12589635	2021-04-28	B.1.1.7	Alpha	2B	Definite HAI
86	EPI_ISL_12589636	2021-05-06	B.1.1.7	Alpha	3AB	Probable HAI
87	EPI_ISL_12589637	2021-05-06	B.1.1.7	Alpha	5B	Probable HAI
88	EPI_ISL_12589653	2021-05-14	B.1.1.7	Alpha	5B	Probable HAI
89	EPI_ISL_12589638	2021-05-17	B.1.1.7	Alpha	5D	Healthcare worker
90	EPI_ISL_12589639	2021-08-09	AY.5	Delta	6A	Healthcare worker
91	EPI_ISL_12589583	2021-08-11	AY.43	Delta	3C	Indeterminate HAI
92	EPI_ISL_12589607	2021-08-18	AY.43	Delta	ED	Healthcare worker

93	EPI_ISL_12589585	2021-08-29	AY.33	Delta	1A	Indeterminate HAI
94	EPI_ISL_3987574	2021-08-30	AY.33	Delta	1A	Indeterminate HAI
95	EPI_ISL_12589587	2021-09-03	AY.43	Delta	3A	Healthcare worker
96	EPI_ISL_12610928	2021-09-05	AY.34	Delta	5C	Healthcare worker
97	EPI_ISL_4505355	2021-09-20	B.1.617.2	Delta	4B	Definite HAI
98	EPI_ISL_12589575	2021-10-11	AY.73	Delta	3A	Healthcare worker
99	EPI_ISL_12589534	2021-10-16	AY.73	Delta	4B	Definite HAI
100	EPI_ISL_12589535	2021-10-18	AY.73	Delta	4C	Probable HAI
101	EPI_ISL_5854776	2021-10-19	AY.73	Delta	4C	Healthcare worker
102	EPI_ISL_5945403	2021-10-19	AY.73	Delta	4C	Probable HAI
103	EPI_ISL_12589588	2021-10-20	AY.4.9	Delta	ED	Healthcare worker
104	EPI_ISL_12589584	2021-10-21	AY.43	Delta	1C	Healthcare worker
105	EPI_ISL_12610925	2021-10-24	AY.126	Delta	3C	Indeterminate HAI
106	EPI_ISL_12589595	2021-10-24	AY.43	Delta	ED	Healthcare worker
107	EPI_ISL_12589536	2021-10-26	AY.125	Delta	4D	Indeterminate HAI
108	EPI_ISL_12589550	2021-10-28	AY.43	Delta	3C	Definite HAI
109	EPI_ISL_12610923	2021-10-29	AY.43	Delta	6C	Healthcare worker
110	EPI_ISL_7123504	2021-10-31	AY.43	Delta	2A	Probable HAI
111	EPI_ISL_12589551	2021-10-31	AY.98.1	Delta	6A	Healthcare worker
112	EPI_ISL_12589538	2021-11-01	AY.43	Delta	2A	Probable HAI
113	EPI_ISL_12589553	2021-11-02	AY.43	Delta	3B	Healthcare worker
114	EPI_ISL_12589552	2021-11-02	AY.43	Delta	6A	Probable HAI
115	EPI_ISL_12610921	2021-11-02	AY.98.1	Delta	6A	Healthcare worker
116	EPI_ISL_12610919	2021-11-02	AY.98.1	Delta	Other	Healthcare worker
117	EPI_ISL_12589539	2021-11-03	AY.43	Delta	3C	Definite HAI
118	EPI_ISL_12610918	2021-11-03	AY.98.1	Delta	6A	Healthcare worker
119	EPI_ISL_12589596	2021-11-03	AY.98.1	Delta	6A	Healthcare worker
120	EPI_ISL_12589555	2021-11-08	AY.112	Delta	3B	Healthcare worker
121	EPI_ISL_12589556	2021-11-08	AY.43	Delta	5A	Indeterminate HAI
122	EPI_ISL_12589554	2021-11-08	AY.98.1	Delta	6A	Healthcare worker
123	EPI_ISL_12589557	2021-11-10	AY.4.2	Delta	2D	Healthcare worker
124	EPI_ISL_12610927	2021-11-12	AY.34	Delta	1C	Probable HAI
125	EPI_ISL_12589558	2021-11-12	AY.43	Delta	ED	Healthcare worker
126	EPI_ISL_12589559	2021-11-13	AY.43	Delta	2C	Healthcare worker
127	EPI_ISL_12589540	2021-11-13	AY.43	Delta	2C	Definite HAI
128	EPI_ISL_12589541	2021-11-13	AY.43	Delta	2C	Probable HAI
129	EPI_ISL_12610920	2021-11-15	AY.98.1	Delta	1C	Definite HAI
130	EPI_ISL_12589597	2021-11-15	AY.9.2	Delta	2B	Healthcare worker
131	EPI_ISL_12589542	2021-11-15	AY.43	Delta	2C	Definite HAI
132	EPI_ISL_12589590	2021-11-15	AY.43	Delta	2C	Definite HAI
133	EPI_ISL_12589560	2021-11-15	AY.112	Delta	3A	Healthcare worker
134	EPI_ISL_12610924	2021-11-15	AY.126	Delta	4A	Healthcare worker
135	EPI_ISL_12589598	2021-11-16	AY.122	Delta	1C	Healthcare worker
136	EPI_ISL_12589562	2021-11-17	AY.43	Delta	1C	Healthcare worker
137	EPI_ISL_12589599	2021-11-17	AY.43	Delta	1C	Healthcare worker
138	EPI_ISL_12610926	2021-11-17	AY.126	Delta	2D	Healthcare worker
139	EPI_ISL_12589561	2021-11-17	AY.4.9	Delta	3B	Healthcare worker
140	EPI_ISL_12589564	2021-11-18	AY.43	Delta	2C	Indeterminate HAI

141	EPI_ISL_12589563	2021-11-18	AY.43	Delta	Other	Healthcare worker
142	EPI_ISL_12589600	2021-11-19	AY.133	Delta	Other	Healthcare worker
143	EPI_ISL_12610922	2021-11-19	AY.43	Delta	ED	Healthcare worker
144	EPI_ISL_12589566	2021-11-20	AY.98.1	Delta	1C	Healthcare worker
145	EPI_ISL_6706113	2021-11-21	AY.4	Delta	3B	Healthcare worker
146	EPI_ISL_12589592	2021-11-22	AY.43	Delta	4B	Definite HAI
147	EPI_ISL_12589591	2021-11-22	AY.43	Delta	4B	Probable HAI
148	EPI_ISL_12589649	2021-11-22	AY.4	Delta	6A	Healthcare worker
149	EPI_ISL_12589569	2021-11-22	AY.43	Delta	6B	Healthcare worker
150	EPI_ISL_12589565	2021-11-22	AY.4.9	Delta	Other	Healthcare worker
151	EPI_ISL_12589568	2021-11-22	AY.43	Delta	Other	Healthcare worker
152	EPI_ISL_12589570	2021-11-23	AY.92	Delta	6A	Indeterminate HAI
153	EPI_ISL_12589593	2021-11-24	AY.43	Delta	2C	Definite HAI
154	EPI_ISL_12589543	2021-11-24	AY.43	Delta	2C	Definite HAI
155	EPI_ISL_12589571	2021-11-25	AY.43	Delta	Other	Healthcare worker
156	EPI_ISL_12589572	2021-11-25	AY.4	Delta	Other	Healthcare worker
157	EPI_ISL_12589574	2021-11-26	AY.127	Delta	Other	Healthcare worker
158	EPI_ISL_12589573	2021-11-26	AY.4	Delta	Other	Healthcare worker
159	EPI_ISL_12589576	2021-11-28	AY.43	Delta	4B	Healthcare worker
160	EPI_ISL_12589577	2021-11-29	AY.43	Delta	Other	Healthcare worker
161	EPI_ISL_12589601	2021-11-30	AY.127	Delta	Other	Healthcare worker
162	EPI_ISL_12589578	2021-12-01	AY.113	Delta	3C	Healthcare worker
163	EPI_ISL_12589602	2021-12-01	AY.43	Delta	4B	Healthcare worker
164	EPI_ISL_12589579	2021-12-01	AY.98.1	Delta	Other	Healthcare worker
165	EPI_ISL_12589580	2021-12-02	AY.4.6	Delta	1A	Healthcare worker
166	EPI_ISL_12589605	2021-12-02	B.1.617.2	Delta	4C	Indeterminate HAI
167	EPI_ISL_12589581	2021-12-03	AY.43	Delta	Other	Healthcare worker
168	EPI_ISL_12589582	2021-12-04	AY.4	Delta	4A	Healthcare worker
169	EPI_ISL_12589603	2021-12-05	AY.4	Delta	1B	Indeterminate HAI
170	EPI_ISL_12589544	2021-12-19	AY.43	Delta	4C	Indeterminate HAI
171	EPI_ISL_12589645	2021-12-21	BA.1.18	Omicron	1A	Healthcare worker
172	EPI_ISL_12589545	2021-12-21	AY.92	Delta	4C	Indeterminate HAI
173	EPI_ISL_12589546	2021-12-22	AY.43	Delta	3AB	Definite HAI
174	EPI_ISL_9775157	2021-12-23	AY.122	Delta	3C	Indeterminate HAI
175	EPI_ISL_12589547	2021-12-24	AY.43	Delta	3AB	Indeterminate HAI
176	EPI_ISL_12589644	2021-12-24	BA.1.1	Omicron	4C	Definite HAI
177	EPI_ISL_12589604	2021-12-26	B.1.617.2	Delta	6A	Healthcare worker
178	EPI_ISL_12589548	2021-12-27	AY.43	Delta	3AB	Probable HAI
179	EPI_ISL_9596724	2021-12-27	AY.43	Delta	3AB	Definite HAI
180	EPI_ISL_12589647	2021-12-27	BA.1.1	Omicron	Other	Healthcare worker
181	EPI_ISL_12589549	2021-12-29	AY.43	Delta	3AB	Probable HAI
182	EPI_ISL_12589594	2021-12-31	AY.43	Delta	3AB	Probable HAI
183	EPI_ISL_12589651	2021-12-31	BA.1.18	Omicron	ED	Healthcare worker
184	EPI_ISL_12589640	2022-01-04	AY.98.1	Delta	ED	Healthcare worker
185	EPI_ISL_8622950	2022-01-05	BA.1.1	Omicron	2B	Healthcare worker
186	EPI_ISL_12589621	2022-01-05	BA.1.1	Omicron	ED	Healthcare worker
187	EPI_ISL_8799558	2022-01-06	BA.1	Omicron	2C	Probable HAI
188	EPI_ISL_12589622	2022-01-06	BA.1.14	Omicron	3A	Healthcare worker

189	EPI_ISL_12589643	2022-01-06	BA.1.17.2	Omicron	3AB	Definite HAI
190	EPI_ISL_12589634	2022-01-06	BA.1.14	Omicron	3D	Healthcare worker
191	EPI_ISL_12589632	2022-01-06	BA.1.1	Omicron	ED	Healthcare worker
192	EPI_ISL_12589625	2022-01-08	BA.1.17	Omicron	3A	Healthcare worker
193	EPI_ISL_12589624	2022-01-08	BA.1.1.14	Omicron	ED	Healthcare worker
194	EPI_ISL_8865794	2022-01-11	BA.1.1	Omicron	3AB	Indeterminate HAI
195	EPI_ISL_12589626	2022-01-12	BA.1.1.14	Omicron	1B	Healthcare worker
196	EPI_ISL_8865791	2022-01-12	BA.1.1.1	Omicron	1B	Indeterminate HAI
197	EPI_ISL_12589609	2022-01-14	BA.1.18	Omicron	1C	Healthcare worker
198	EPI_ISL_12589608	2022-01-14	BA.1.1	Omicron	3D	Healthcare worker
199	EPI_ISL_12589610	2022-01-15	BA.1.17.2	Omicron	ED	Healthcare worker
200	EPI_ISL_9351882	2022-01-16	BA.1.1	Omicron	3C	Indeterminate HAI
201	EPI_ISL_9351883	2022-01-17	BA.1.18	Omicron	1C	Probable HAI
202	EPI_ISL_12589628	2022-01-17	BA.1.14	Omicron	3B	Healthcare worker
203	EPI_ISL_12589611	2022-01-17	BA.1.18	Omicron	3B	Healthcare worker
204	EPI_ISL_9351884	2022-01-17	BA.1.1	Omicron	3C	Probable HAI
205	EPI_ISL_9351885	2022-01-17	BA.1.1	Omicron	3C	Indeterminate HAI
206	EPI_ISL_12589652	2022-01-17	BA.1.1	Omicron	ED	Healthcare worker
207	EPI_ISL_12589648	2022-01-18	BA.1.1	Omicron	5B	Healthcare worker
208	EPI_ISL_9356641	2022-01-20	BA.1	Omicron	3AB	Definite HAI
209	EPI_ISL_12589631	2022-01-20	BA.1.1.1	Omicron	3C	Healthcare worker
210	EPI_ISL_9356639	2022-01-20	BA.1.1	Omicron	3C	Definite HAI
211	EPI_ISL_9356640	2022-01-20	BA.1.1	Omicron	3C	Probable HAI
212	EPI_ISL_9356639	2022-01-20	BA.1.1	Omicron	4B	Definite HAI
213	EPI_ISL_12589612	2022-01-21	BA.1	Omicron	3C	Healthcare worker
214	EPI_ISL_12589613	2022-01-21	BA.1.1	Omicron	3C	Healthcare worker
215	EPI_ISL_12589615	2022-01-22	BA.1.14	Omicron	2B	Healthcare worker
216	EPI_ISL_12589614	2022-01-22	BA.1.1	Omicron	2C	Healthcare worker
217	EPI_ISL_12589642	2022-01-22	BA.1.18	Omicron	2D	Healthcare worker
218	EPI_ISL_9351967	2022-01-23	BA.1.1	Omicron	1B	Healthcare worker
219	EPI_ISL_12589616	2022-01-23	BA.1.1	Omicron	3A	Healthcare worker
220	EPI_ISL_9351968	2022-01-24	BA.1.15.1	Omicron	1B	Healthcare worker
221	EPI_ISL_12589627	2022-01-24	BA.2.10	Omicron	3A	Healthcare worker
222	EPI_ISL_12589641	2022-01-24	BA.1.1	Omicron	6C	Healthcare worker
223	EPI_ISL_9500555	2022-01-25	BA.1	Omicron	1C	Healthcare worker
224	EPI_ISL_9500041	2022-01-26	BA.1.1	Omicron	3B	Healthcare worker
225	EPI_ISL_9500043	2022-01-26	BA.1.1	Omicron	Other	Healthcare worker
226	EPI_ISL_12589618	2022-01-27	BA.1.1	Omicron	1B	Healthcare worker
227	EPI_ISL_12589617	2022-01-27	BA.1.1	Omicron	3C	Healthcare worker
228	EPI_ISL_9596646	2022-01-27	BA.1.1	Omicron	4A	Probable HAI
229	EPI_ISL_9500419	2022-01-27	BA.1.1	Omicron	Other	Healthcare worker
230	EPI_ISL_9596649	2022-01-29	BA.1.1	Omicron	2B	Healthcare worker
231	EPI_ISL_9596625	2022-01-31	BA.1.1	Omicron	3D	Definite HAI
232	EPI_ISL_9596626	2022-01-31	BA.1.14	Omicron	3D	Probable HAI
233	EPI_ISL_12589650	2022-01-31	BA.1.15.1	Omicron	4C	Probable HAI
234	EPI_ISL_12589619	2022-01-31	BA.1.1.1	Omicron	ED	Healthcare worker
235	EPI_ISL_12589633	2022-01-31	BA.1.1	Omicron	6C	Healthcare worker
236	EPI_ISL_9596624	2022-01-31	BA.1.1	Omicron	1B	Healthcare worker

237	EPI_ISL_9596628	2022-01-31	BA.1.1	Omicron	6A	Healthcare worker
238	EPI_ISL_10334153	2022-02-01	BA.1	Omicron	3AB	Probable HAI
239	EPI_ISL_9775113	2022-02-01	BA.1.17	Omicron	3AB	Indeterminate HAI
240	EPI_ISL_9775115	2022-02-01	BA.1.1	Omicron	4B	Probable HAI
241	EPI_ISL_9775110	2022-02-01	BA.1.1.14	Omicron	Other	Healthcare worker
242	EPI_ISL_9775113	2022-02-01	BA.2	Omicron	6C	Healthcare worker
243	EPI_ISL_9775133	2022-02-02	BA.1.1	Omicron	Other	Healthcare worker
244	EPI_ISL_9775134	2022-02-02	BA.1.1	Omicron	3D	Healthcare worker
245	EPI_ISL_9860863	2022-02-04	BA.1.14	Omicron	1A	Indeterminate HAI
246	EPI_ISL_9860862	2022-02-04	BA.1.18	Omicron	ED	Healthcare worker
247	EPI_ISL_9860876	2022-02-07	BA.1.15.1	Omicron	4C	Probable HAI
248	EPI_ISL_9860878	2022-02-07	BA.1.1	Omicron	6A	Healthcare worker
249	EPI_ISL_10334127	2022-02-08	BA.1.1	Omicron	3B	Healthcare worker
250	EPI_ISL_10334137	2022-02-10	BA.1.1.1	Omicron	2C	Healthcare worker
251	EPI_ISL_10334148	2022-02-12	BA.1.1	Omicron	Other	Healthcare worker
252	EPI_ISL_10334150	2022-02-13	BA.2	Omicron	1B	Healthcare worker
253	EPI_ISL_10334155	2022-02-15	BA.1.1	Omicron	ED	Healthcare worker
254	EPI_ISL_12589620	2022-02-15	BA.1.1.1	Omicron	ED	Healthcare worker
255	EPI_ISL_10334161	2022-02-15	BA.1.1	Omicron	4B	Healthcare worker
256	EPI_ISL_12589606	2022-02-16	BA.2	Omicron	ED	Healthcare worker
257	EPI_ISL_10334158	2022-02-16	BA.2	Omicron	1A	Healthcare worker
258	EPI_ISL_10590273	2022-02-22	BA.1.1	Omicron	4C	Healthcare worker
259	EPI_ISL_10590275	2022-02-22	BA.1.1	Omicron	ED	Healthcare worker
260	EPI_ISL_10794399	2022-02-24	BA.1.1	Omicron	Other	Healthcare worker
261	EPI_ISL_10794405	2022-02-28	BA.1.1	Omicron	1C	Healthcare worker
262	EPI_ISL_10843946	2022-03-02	BA.1.1	Omicron	3A	Healthcare worker
