# Supplementary material for: Healthcare-Associated COVID-19 across Five Pandemic Waves: Prediction Models and Genomic Analyses
Source: Viruses. 2022 Oct 18;14(10):2292. doi: 10.3390/v14102292 (PMC9607632; doi:10.3390/v14102292)
Supplement: Supplementary file 1 [file viruses-14-02292-s001.zip › Supplementary Table S2.pdf]

Supplementary Table S2

Demographic, clinical and laboratory data of symptomatic healthcare-associated versus community-associated hospitalised COVID-19 patients (UZ Brussel, 2020-22): univariate analysis

Patient demographics		Healthcare-associated (N = 165)	%	Community-associated (N = 159)	%	p-value
Gender						0.658
	Male	83	50.3	84	52.8	
	Female	82	49.7	75	47.2	
Age (years)		74.0 (63.5 - 82.0)		67.0 (53.0 - 80.0)		0.0012
Smoking	Never	62	52.1	71	61.2	0.321
	Former	39	32.8	33	28.4	
	Current	18	15.1	12	10.3	
BMI (kg / m²)		24.7 (22.09 - 29.7)		27.3 (24.2 - 31.6)		0.0004
Frailty						< 0.0001
	1	2	1.4	21	14.2	
	2	6	4.2	28	18.9	
	3	21	14.8	39	26.4	
	4	28	19.7	27	18.2	
	5	44	31.0	11	7.4	
	6	21	14.8	11	7.4	
	7	17	12.0	8	5.4	
	8	3	2.1	1	0.7	
	9	0	0.0	2	1.4	
**Hospitalization**						
ICU admission during stay		45	28.0	34	21.4	0.196
Deceased		44	27.3	20	12.6	0.0012
Stay from COVID-19 diagnosis (days)		12.5 (7.0 - 21.8)		8.0 (5.0 - 12.0)		< 0.0001
**Symptoms at COVID-19 diagnosis**						
Fever		81	54.0	90	59.6	0.353
Chills		22	37.9	36	62.1	0.169
Cough		81	59.6	108	73.0	0.0174
Shortness of breath		66	47.1	111	74.5	< 0.0001
Tachypnoea		59	40.4	88	59.5	0.0016
Headache		10	9.3	36	29.5	0.0001
Sore throat		11	11.3	11	10.0	0.823
Coryza		10	10.8	18	16.8	0.229
Malaise		39	39.0	76	64.4	0.0002
Diarrhoea		26	20.6	41	29.1	0.122
Nausea		26	21.7	43	30.9	0.121
Vomiting		21	16.7	28	20.0	0.529
Ageusia		2	2.2	17	17.9	0.0004
Anosmia		5	5.4	19	19.2	0.004
**Laboratory parameters at COVID-19 diagnosis**						
Trombocytes (x 10^6 / µL)		243.0 (180.0 - 317.0)		190.0 (158.0 - 266.0)		0.0011
Leucocytes (x 10^3 / µL)		6.1 (4.7 - 8.6)		6.5 (4.5 - 11.2)		0.396
Neutrophil / lymphocyte ratio		4.9 (2.7 - 7.9)		4.9 (3.2 - 9.6)		0.534
D-dimers (ng / mL)		1312.0 (676.0 - 2468.0)		997.0 (620.5 - 1823.0)		0.051
Ferritin (µg / L)		597.0 (277.5 - 1074.0)		547.0 (225.0 - 1006.0)		0.557
CRP (mg / L)		36.7 (16.5 - 75.9)		58.9 (23.2 - 136.2)		0.0083
**Comorbidities of COVID-19 patients**						
Anaemia		35	22.2	15	9.9	0.0034
Asthma		13	8.9	12	8.3	1
Cancer		41	26.3	14	9.3	0.0002
Hypertension		91	58.7	69	45.7	0.0295
Dementia		20	13.3	7	4.9	0.0149
Diabetes		42	27.1	46	30.5	0.53
Heart disease		66	43.1	36	24.3	0.0006
Liver disease		10	6.4	4	2.7	0.171
Neuromuscular disease		12	8.0	4	2.8	0.0702
Renal disease		43	27.4	16	10.5	0.0002

Univariate statistical analysis of continuous data, represented as median (IQR), was performed using unpaired t-test or Mann-Whitney test (depending on normal distribution of data). Univariate statistical analysis of categorical data was done with Fisher's exact test. P < 0.05 was considered as statistically significant. (BMI – body mass index, ICU – intensive care unit, CRP – C-reactive protein)
