# Supplementary material for: Healthcare-Associated COVID-19 across Five Pandemic Waves: Prediction Models and Genomic Analyses
Source: Viruses. 2022 Oct 18;14(10):2292. doi: 10.3390/v14102292 (PMC9607632; doi:10.3390/v14102292)
Supplement: Supplementary file 1 [file viruses-14-02292-s001.zip › Supplementary Table S3.pdf]

Supplementary Table S3

Hospital-acquired infections, according to the ECDC case-source definition

**Case source COVID-19**	**Total number (% of total HAI)**	**Number sequenced (% of category)**	**% belonging to a genome cluster**
Indeterminate HAI	93 (34.6)	36 (38.7)	70.3
Probable HAI	86 (32.0)	40 (46.5)	87.8
Definite HAI	90 (33.5)	47 (52.2)	90.0
